# Improving Mechanical, Electrical and Thermal Properties of Fluororubber by Constructing Interconnected Carbon Nanotube Networks with Chemical Bonds and F–H Polar Interactions

**DOI:** 10.3390/polym14224989

**Published:** 2022-11-17

**Authors:** Yurou Chen, Yadong Wu, Jun Li, Xuqiang Peng, Shun Wang, Huile Jin

**Affiliations:** 1 Wenzhou Key Lab of Advanced Energy Storage and Conversion, Zhejiang Province Key Lab of Leather Engineering, College of Chemistry and Materials Engineering, Wenzhou University, Wenzhou 325035, China; 2 Institute of New Materials and Industrial Technologies, Wenzhou 325024, China

**Keywords:** fluororubber, aminated carbon nanotubes, acidified carbon nanotubes, mechanical properties, interfacial interactions

## Abstract

To improve the properties of fluororubber (FKM), aminated carbon nanotubes (CNTs-NH_2_) and acidified carbon nanotubes (CNTs-COOH) were introduced to modulate the interfacial interactions in FKM composites. The effects of chemical binding and F–H polar interactions between CNTs-NH_2_, CNTs-COOH, and FKM on the mechanical, electrical, thermal, and wear properties of the FKM composites were systematically investigated. Compared to the pristine FKM, the tensile strength, modulus at 100% strain, hardness, thermal conductivity, carbon residue rate, and electrical conductivity of CNTs-NH_2_/CNTs-COOH/FKM were increased by 112.2%, 587.5%, 44.2%, 37.0%, 293.5%, and nine orders of magnitude, respectively. In addition, the wear volume of CNTs-NH_2_/CNTs-COOH/FKM was reduced by 29.9%. This method provides a new and effective way to develop and design high-performance fluororubber composites.

## 1. Introduction

In recent years, a rubber material that can withstand strong corrosion and high temperatures in a harsh environment is urgently needed in the petrochemical, automotive, and aerospace fields [1,2,3]. The copolymer consisting of hexafluoropropylene, vinylidene fluoride, and tetrafluoroethylene with a cure site monomer, constituting a special polymer material with a large number of fluorine atoms in its structure, has an incredibly high resistance to chemical media and can be used for a long time under a high temperature of 250 °C [4]. However, it still suffers from poor mechanical and electrical properties and inadequate wear resistance [5,6]. In order to overcome these problems, researchers have proposed enhancing the performance of fluororubber (FKM) by the filler modification method.

Carbon nanotubes (CNTs) are widely used in rubber modification due to their excellent reinforcing, thermal, and electrical properties [7,8,9,10]. With the wide application of CNTs in FKM, the enhancement effect of modified CNTs on FKM has gradually attracted the attention of researchers [11,12,13]. Heidarian et al. [14] found that the hydrogen bonding and compatibility between acidified carbon nanotubes (CNTs-COOH) and FKM are stronger compared to CNTs in their research work. Meanwhile, a double cross-linked network formed between aminated carbon nanotubes (CNTs-NH_2_) and FKM was reported by Gao et al. [15]. It was demonstrated by experimental data that the covalent bond (C=N bond) existing between CNTs-NH_2_ and FKM effectively enhanced the thermal conductivity and tensile properties of FKM by 17.1% and 65.8% compared to CNTs-COOH. In addition, similar results were found in the research work of Yang et al. [16]. As a result, an increase in the cross-link density or type of interaction force can significantly improve the properties of fluororubber. According to the previous work [17], we found that this method is also applicable to the two-filler system. Therefore, we needed to find a filler that could interact with both CNTs-NH_2_ and FKM to improve the performance of nanocomposites more effectively. Among the types of polar interactions, non-covalent bonds, including hydrogen bonds, π–π bonds, and so on, have great advantages in modulating interfacial interactions without strict reaction conditions [18,19].

Thus, in this contribution, we propose a method for enhancing the mechanical, thermal, and electrical properties of FKM by forming F–H polar interactions and C=O bonds with FKM and CNTs-NH_2_ through reactive groups (-COOH) on the surface of CNTs-COOH. This method not only has a facile preparation process but can also effectively improve the interfacial interaction between the filler and the FKM matrix. In addition, the wear resistance of the FKM composites was systematically investigated. This new approach promises greater competitive advantages in high-temperature, wear-resistant-sealing, and battery separator applications.

## 2. Materials and Methods

### 2.1. Materials

The material used was Viton GF-600S fluororubber, which is a terpolymer comprising hexafluoro-propylene (HFP), vinylidene fluoride (VDF), and tetra-fluoroethylene (TFE). The aminated multiwalled carbon nanotubes (CNTs-NH_2_, purity > 98%, and with an amino content of 0.7 mmol/g) and carboxylated multiwalled carbon nanotubes (CNTs-COOH, purity > 98%, with a carboxyl content of 1 mmol/g) were obtained from Shenzhen Suiheng RGO Technology Co., Ltd. (Shenzhen, China). The multiwalled carbon nanotubes were purchased from Jiangsu Cnano Technology Co., Ltd. (Zhenjiang, China). In addition, vulcanizing agents, accelerators, and acid absorbers, e.g., 2,5-dimethyl-2,5-bis (tert-butylperoxy) hexane (Trigonox^®^ 101-50D), triallyl isocyanurate (TAIC), and ZnO, were supplied by Yuyao Mingri Chemical Co., Ltd. (Yuyao, China), Rhein Chemical (Qingdao, China) Co., Ltd. (Qingdao, China), and Yangzhou Liuxin Zinc Products Co., Ltd. (Yangzhou, China), respectively.

### 2.2. Preparation of CNTs-NH_2_/CNTs-COOH/FKM Composite

**Figure 1:**
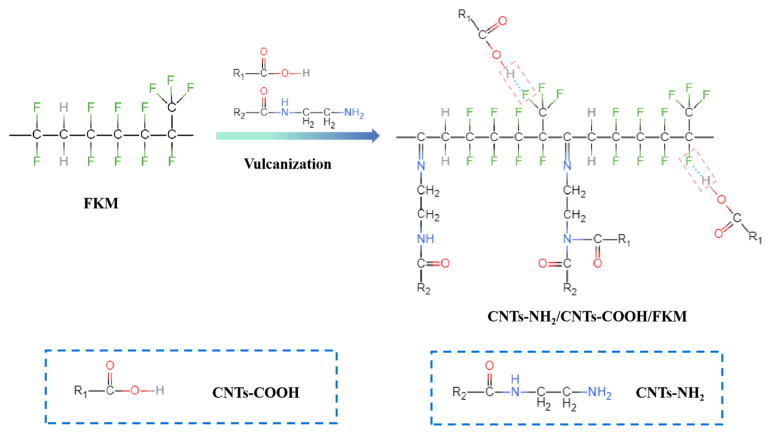
The reaction mechanism of CNTs-NH_2_/CNTs-COOH/FKM composite.

Mixing the pristine FKM with pre-blended ZnO and TAIC was performed using a mixer (KHB8, Guangdong Lina Industrial Co., Ltd., Dongguan, China) with a roll temperature of 50 °C. After the FKM was rolled and cut three times from each side of the mill, CNTs, CNTs-COOH, and CNTs-NH_2_ were fed into the mill. Trigonox^®^ 101-50D was added in sequence and then rolled and cut 6 times on the open mill or until it became homogenized. After 24 h, re-milling was performed with a roll temperature of 30 °C. The rubber sheet was pressed with a flat vulcanizing machine (XH-406B, Xihua Testing Instrument Co., Ltd., Dongguan, China) at 177 °C under a pressure of 10 MPa for 7 min. Finally, the vulcanizate was post-cured at 230 °C for 2 h, and the as-prepared sheet was denoted as CNTs-NH_2_/CNTs-COOH/FKM composite. The reaction mechanism of CNTs-NH_2_/CNTs-COOH/FKM nanocomposites is shown in Figure 1.

In order to compare the effects of different interaction forces on their properties, FKM, CNTs-COOH/FKM, and CNTs-NH_2_/FKM were also prepared in this study according to a similar procedure, and their corresponding formulations are listed in Table 1.

### 2.3. Characterization

Microstructures of the nanofillers and the fracture surface morphology of different FKM nanocomposites after tensile test were examined by scanning electron microscopy (SEM, Nova 200 NanoLab, Hillsboro, OR, USA) at an accelerating voltage of 5 kV.

The functional groups of MWCNT-A and RGO were examined using Fourier transform infrared spectroscopy (FTIR, Spotlight 200i, Perkin Elmer, Waltham, MA, USA) with a wavenumber range of 400–4000 cm^−1^ and a resolution of 2 cm^−1^.

A total of 30–50 mg of post-cured sample was subjected to TGA runs from 40 °C to 600 °C. This was carried out on a PerkinElmer thermal analysis system (Pyris 1 TGA, Perkin Elmer, Waltham, MA, USA) at a scan rate of 10 °C/min in a nitrogen atmosphere.

The tensile properties of different FKM nanocomposites were measured using a universal material-testing machine (Instron 5982, Instron, Boston, MA, USA) according to GB/T 528-2009 at a crosshead speed of 300 mm/min. The shore A hardness was measured by using an LX-A sclerometer (JLX-A, Qingbo, China) according to ASTM D 2240.

The electrical conductivity of different FKM nanocomposites was measured by using a digital ultra-high resistance microcurrent-measuring instrument (EST121, EST, Beijing, China) at a voltage of 220 V. The electrical conductivity value was calculated according to Formula (1)

(1) $$\sigma =\frac{1}{\frac{21.24}{d}\times Rv}$$

where *σ* is the conductivity (S/cm), d is the thickness of the sample (cm), and *Rv* is the volume resistance of the sample (Ω). The dimensions of the specimen were 18 mm × 18 mm × 2 mm, and each test was performed on five specimens.

The thermal conductivities of different FKM nanocomposites were tested via a thermal conductivity tester (TC 3000, Xiatech Instrument Factory, Xi’an, China) using a transient hot-wire method at 20 °C. The circular specimens were prepared with a diameter of 30 mm and a thickness of about 2 mm.

A DIN Abrasion Resistance Tester (GT-7012-D, Gotech Testing Machines, Dongguan, China) was used to measure the mass abrasion of nanocomposites. The volumetric wear consumption of nanocomposites was calculated by Formula (2)

(2) $$A=\frac{\Delta m\times 200}{Q\times S}$$

where *A* is the wear volume (mm^3^), Δ*m* is the mass abrasion (mg), *Q* is the abrasion of standard rubber (mg), and *S* is the specific gravity of the sample (mg/mm^3^).

## 3. Results and Discussion

### 3.1. Morphology of CNTs, CNTs-COOH, and CNTs-NH_2_

**Figure 2:**
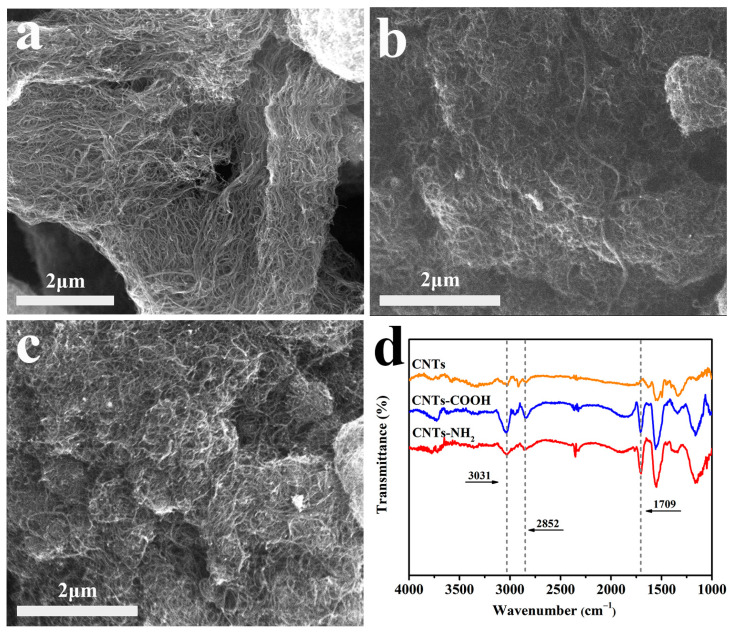
The SEM images of (**a**) CNTs, (**b**) CNTs-COOH, and (**c**) CNTs-NH_2_; (**d**) the FTIR spectra.

The microscopic morphologies of several carbon materials are shown in Figure 2. All three types of carbon nanotubes have a long, thin, and linear shape. In addition, we can clearly observe the strong agglomeration effect and mutual entanglement in the unpredispersed carbon nanotubes.

### 3.2. Chemical Composition Analysis of CNTs, CNTs-COOH, and CNTs-NH_2_

As shown in Figure 2d, the microstructure of the carbon nanotubes has been analyzed by FTIR spectra. The intense peaks at 1709 cm^−1^ for CNTs-COOH and CNTs-NH_2_ can be assigned to the stretching vibration of the C=O of -COOH groups on CNTs-COOH and the C=O of -CONH- groups on CNTs-NH_2_ [15,20]. The bands at 2852 cm^−1^ and 3031 cm^−1^ correspond to the C-H skeleton vibration [21].

### 3.3. Morphology of Different Composites

**Figure 3:**
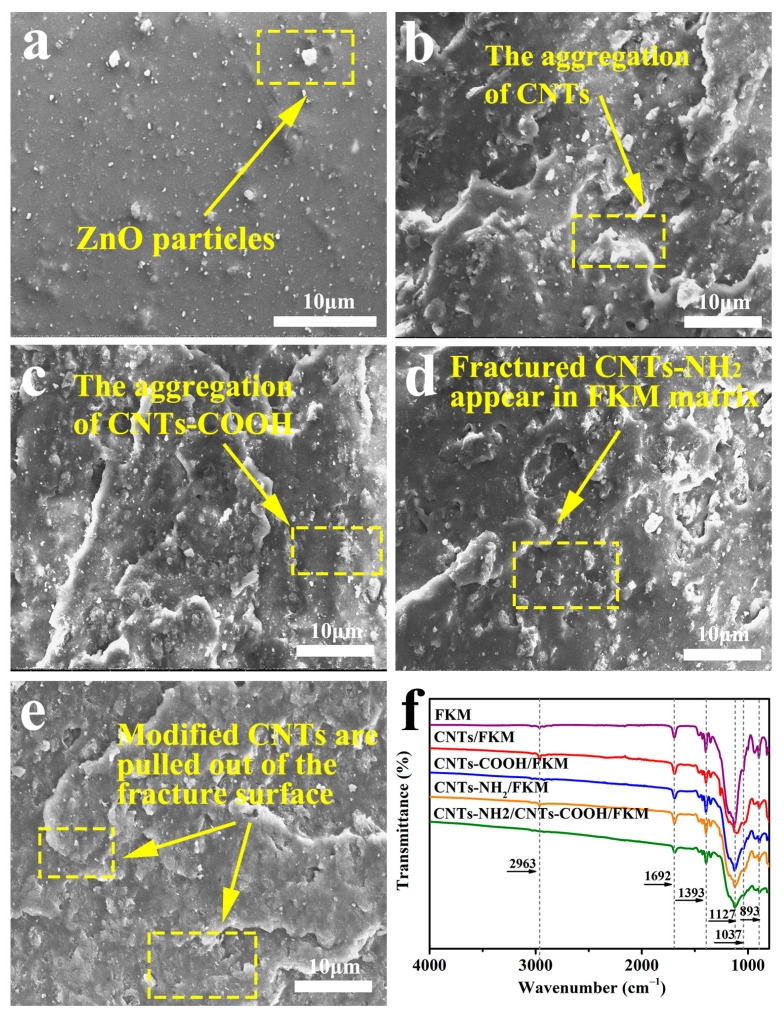
The SEM images of fractured surfaces of (**a**) FKM, (**b**) CNTs/FKM, (**c**) CNTs-COOH/FKM, (**d**) CNTs-NH_2_/FKM, and (**e**) CNTs-NH_2_/CNTs-COOH/FKM; (**f**) the FTIR spectra of different composites.

Figure 3 shows the SEM images of the fracture surfaces of different composites. We can observe the microscopic morphologies of FKM, CNTs/FKM, CNTs-COOH/FKM, CNTs-NH_2_/FKM, and CNTs-NH_2_/CNTs-COOH/FKM. In the red box in Figure 3a, we can see the larger ZnO particles appearing on the surface of the fluororubber. As shown in the red boxes of Figure 3b,c, we can observe partial agglomerations of the carbon nanotubes, similar to those depicted in Figure 2. After the composites were pulled off by external force, some carbon nanotubes were extracted and exposed in the fracture surfaces (Figure 3d,e).

### 3.4. Chemical Composition Analysis of FKM Nanocomposites

To qualitatively analyze the chemical structures of the substances, we used FTIR spectra for the characterization of the composites. In Figure 3, we can see that several composites have absorption peaks of different intensities at 893 cm^−1^, 1037 cm^−1^, 1127 cm^−1^, 1393 cm^−1^, 1692 cm^−1^, and 2963 cm^−1^, which correspond to the -CF_3_ group, C-N bond, -CF_2_-group, -CF-group, C=C bond, and C-H absorption peaks of fluororubbers [22,23]. Among them, the non-conjugated C=C bond at 1692 cm^−1^ can tentatively prove the occurrence of the defluorination hydrogenation reaction as well as the oxidation reaction during the vulcanization process [23]. The peak area ratio of C-N bonds to -CF_3_ (A_C-N_/A_-CF3_) can be calculated by using the -CF_3_ absorption peak, which is not involved in the reaction in fluororubber, as the reference peak; thus, it was derived that the A_C-N_/A_-CF3_ of CNTs-NH_2_/FKM and CNTs-NH_2_/CNTs-COOH/FKM are 0.38 and 0.65, respectively. This indicates that the content of the C-N bond in CNTs-NH_2_/CNTs-COOH/FKM has increased. This proves that the amino group of CNTs-NH_2_ and the carboxyl group of CNTs-COOH chemically reacted during the vulcanization process.

### 3.5. Mechanical Properties

**Figure 4:**
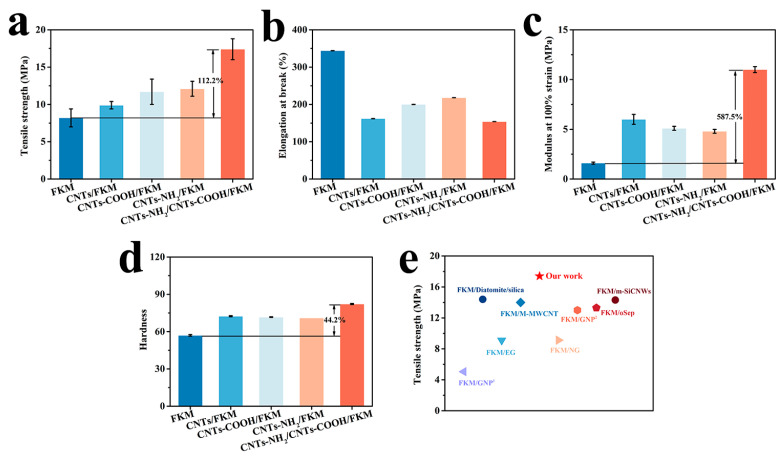
Mechanical properties of nanocomposites: (**a**) tensile strength, (**b**) elongation at break, (**c**) modulus at 100% strain, (**d**) hardness. (**e**) Comparison of the tensile strength of our work with other literatures, including FKM/GNP^1^ [25], FKM/Diatomite/silica [26], FKM/EG [27], FKM/M-MWCNT [16], FKM/NG [28], FKM/GNP^2^ [29], FKM/oSep [30] and FKM/m-SiCNWs [2].

Figure 4 shows the mechanical properties of FKM, CNTs/FKM, CNTs-COOH/FKM, CNTs-NH_2_/FKM, and CNTs-NH_2_/CNTs-COOH/FKM at 25 °C, including their tensile strength, elongation at break, modulus at 100% strain, and hardness. As shown in Figure 4a, the tensile strength of the pristine FKM is 8.2 MPa. With the addition of CNTs, CNTs-COOH, and CNTs-NH_2,_ the tensile strength of the different FKM composites improved; the reason could be ascribed to the honeycomb network structure formed by the linear CNTs in the FKM matrix, which causes the FKM composites to need a greater external force to produce deformation [5]. In particular, CNTs-NH_2_ can form a chemical cross-linking bond, i.e., a C=N bond, with the molecular chain of the fluororubber [15], while CNTs-COOH can form hydrogen bonds and C-N bonds with fluororubber and CNTs-NH_2_, respectively. Therefore, when the two modified carbon nanotubes were added to FKM at the same time, the tensile strength of CNTs-NH_2_/CNTs-COOH/FKM reached 17.4 MPa and increased by 112.2% compared with the pristine FKM. This can be interpreted as the network structure of the linear material and the interaction force formed between the fillers and the fluororubber causing the network structure inside the FKM to be less susceptible to damage, thus improving the tensile strength of CNTs-NH_2_/CNTs-COOH/FKM.

The elongation at break and modulus at 100% strain of the modified FKM composites are reported in Figure 4b,c. Due to its own rigid qualities, the addition of carbon nanotubes makes the fluororubber much more rigid, thus lowering the elongation at break [16,24]. Therefore, the elongation at break of fluororubber was reduced to different degrees when three different modified carbon nanotubes were added to FKM alone. When both CNTs-COOH and CNTs-NH_2_ were added to fluororubber, the decrease in the elongation at break was comparable to that of CNTs/FKM. This can be explained by the fact that the interaction between the fillers and the rubber causes the molecular chains to grow, but at the same time, the increase in the cross-link density prevents the molecular chains from slipping easily, thus causing a decrease in the elongation at break. The results in Figure 4c support the above findings. In addition, the modulus at 100% strain of fluororubber with the addition of two fillers increased by 587.5%. Since the presence of reactive groups on the surface of CNTs leads to the growth of molecular chains in the FKM matrix, the fluororubber containing modified CNTs experiences lower tensile stress when subjected to an equivalent amount of external force. Meanwhile, the interactions in CNTs-NH_2_/CNTs-COOH/FKM not only led to an increase in molecular weight and cross-link density, but also relatively reduced the adverse effect of the free ends, thus improving its performance. It has been further demonstrated that increasing the interaction force between the filler and fluororubber is necessary to improve their tensile properties.

The hardness of different FKM composites was tested and the results are shown in Figure 4d. It can be seen that the hardness of the pristine FKM was only 57. With the addition of CNTs, CNTs-COOH, and CNTs-NH_2,_ the hardness of all the different FKM composites improved. However, the hardness of the CNTs/FKM, CNTs-COOH, and CNTs-NH_2_/FKM/FKM composites did not show a significant difference. Whereas after the addition of CNTs-COOH and CNTs-NH_2_ at the same time, the hardness of CNTs-NH_2_/CNTs-COOH/FKM was increased by 44.2% relative to the raw rubber. This is related to the strength of carbon nanotubes and the formation of multiple interactions.

### 3.6. Wear Resistance

**Figure 5:**
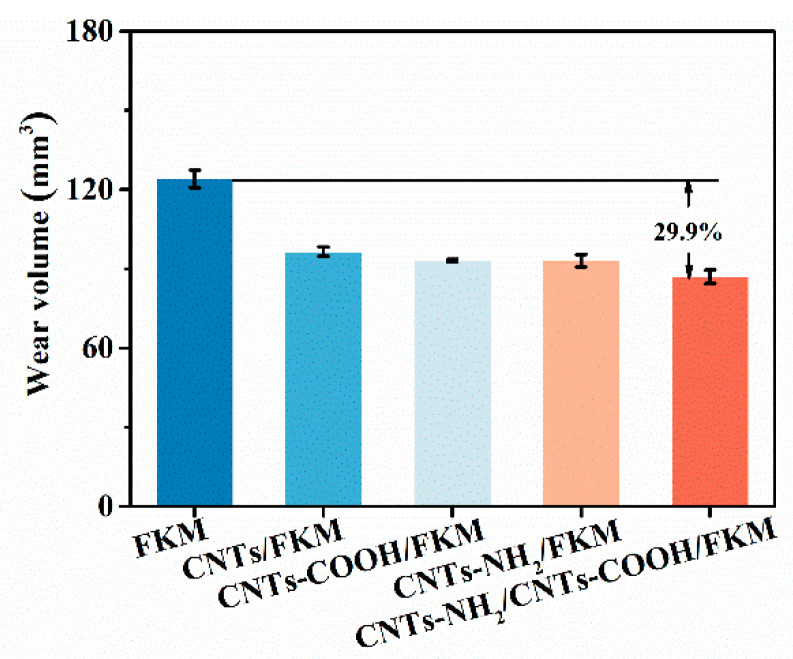
Wear volume of nanocomposites.

The wear resistance of fluororubber seals has a direct influence on their service life in industrial applications. In addition, the cross-link density, self-lubricating properties, and its own strength can improve the wear resistance of fluororubber. As one-dimensional nanomaterials with high strength and stiffness, carbon nanotubes can directly improve the wear resistance of fluororubber. The wear volumes of different fluororubber composites are shown in Figure 5. From the figure, it is clear that the wear volume of FKM shows a gradual decrease with the enhancement of interfacial interactions. In addition, the wear volume of raw rubber was 124.1 mm^3^. When CNTs-NH_2_ and CNTs-COOH were added to fluororubber simultaneously, the wear volume of CNTs-NH_2_/CNTs-COOH/FKM was reduced by 29.9%. This is because the chemical bonds and H–F hydrogen bonds formed by the two carbon materials and the fluororubber molecular chains, respectively, increased the cross-linked species and cross-linked density of the composites, which resulted in lower volumetric wear under wear conditions.

### 3.7. Electrical Properties

**Figure 6:**
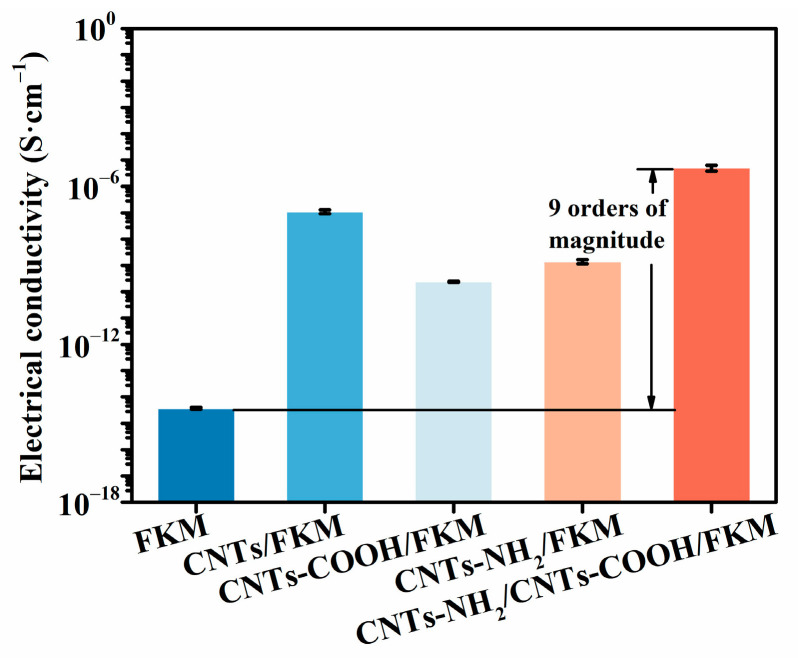
The electrical conductivity of FKM nanocomposites.

To research the effect of several carbon nanotubes on the electrical conductivity of the FKM composites, the electrical conductivity of different FKM nanocomposites was tested by a digital ultra-high resistance microcurrent-mearing instrument, and the results are shown in Figure 6. The conductivity of the pristine FKM was 3.7 × 10^−15^ S/cm. The addition of the linear carbon material formed a conductive network in the fluororubber matrix to improve its conductivity. However, the carbon nanotubes increased their own defects after their modification by the acid treatment, thus reducing their electrical conductivity. In addition, when treated with an amine modification, the conductivity of the carbon nanotubes decreased, but they were still able to adequately improve their conductivity compared with CNTs-COOH. Meanwhile, CNTs-NH_2_ was able to form a chemical bond with FKM using itself as the cross-linking center, which constituted an effective conductive network [15]. On this basis, the addition of CNTs-COOH completed the conductive network of FKM and improved the conductivity of the CNTs-NH_2_/CNTs-COOH/FKM composite by nine orders of magnitude compared with the pristine FKM. We find that this method of establishing a double-network model of crosslinking and conductive networks can stabilize the conductive network, which is consistent with the results reported in the literature [31].

### 3.8. Thermal Properties

**Figure 7:**
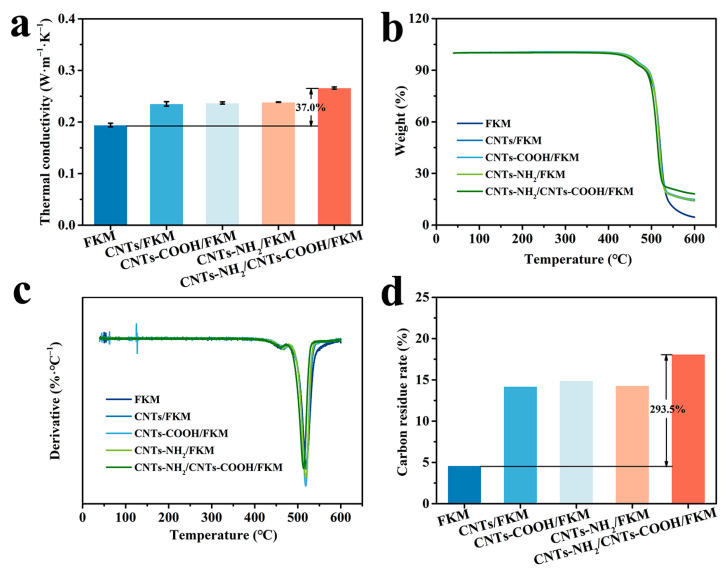
(**a**) Thermal conductivity, (**b**) TG, (**c**) DTG, and (**d**) carbon residue rate of nanocomposites.

The thermal conductivity, TG, DTG, and carbon residue rate of the different composites are shown in Figure 7. From Figure 7a, it can be seen that the thermal conductivity networks formed by these three carbon nanotubes in the fluororubber matrix effectively enhanced the thermal conductivity of fluororubber. When CNTs-NH_2_ and CNTs-COOH were added simultaneously, the thermal conductivity of the CNTs-NH_2_/CNTs-COOH/FKM composite (0.2660 W·m^−1^K^−1^) was improved by 37.0% compared with the pristine fluororubber (0.1942 W·m^−1^K^−1^). This is attributed to the incorporation of highly thermal conductive carbon materials and their formation of a three-dimensional thermal conductive network within the fluororubber. This greatly improves the thermal conductivity pathway within the fluororubber, thus enhancing the fluororubber’s thermal conductivity.

Figure 7b,c show the TG and DTG of several composites. From these two figures, we can glean that the initial decomposition temperatures of the several composites are 437.6 °C, 444.3 °C, 444.3 °C, 442.8 °C, and 435.6 °C. Furthermore, we can find that the addition of a moderate number of carbon fillers can improve the thermal stability of fluororubber. This is because the carbon nanotubes have good heat conductivity and form a thermally conductive network with the molecular chains of fluororubber, which can rapidly transfer the external heat to the interior of the polymer and disintegrate the structure of the polymer in advance. When the thermal network formed by the carbon fillers is improved, the thermal performance of the composite will be affected and degraded. Whereas when the thermal stability of the carbon fillers is superior, the thermal properties of the composite will be improved. Therefore, after the addition of 5 phr CNTs-NH_2_ and 5 phr CNTs-COOH, the thermal network is superior and the thermal stability of the fluororubber decreases.

The carbon residue rate of the five composites at 600 °C are shown in Figure 7d. The carbon residue rate of pristine FKM was only 4.6%. After the addition of carbon materials, the carbon residue rate was significantly increased. By combining the above thermal conductivity and thermal stability, the higher thermal conductivity of CNTs-NH_2_/CNTs-COOH/FKM can accelerate the process of carbon formation and form a dense carbon layer on the surface of the specimen faster than several other composites, improving the carbon residue rate of fluororubber.

## 4. Conclusions

In this study, we reported the enhancement of a carbon nanotube network based on chemical binding and F–H polar interactions in nanocomposites. The experimental results show that the mechanical, electrical, thermal, and wear resistance properties of the composites are greatly improved by the modulation of the interfacial interactions in the composites. The tensile strength, modulus at 100% strain, and hardness of CNTs-NH_2_/CNTs-COOH/FKM were increased to 17.4 MPa, 11.0 MPa, and 82.2, which were 112.2%, 587.5%, and 44.2% higher compared to the pristine FKM. The reason is that the cellular network structure formed by CNTs-NH_2_ and CNTs-COOH in the FKM matrix restricts the deformation of molecular chains. In addition, the chemical binding and F–H polar interactions make the composites better able to absorb stress without being easily damaged when subjected to external forces, thus enhancing their tensile strength, modulus at 100% strain, and hardness. However, the reduced deformation of the molecular chains apparently decreases the composites’ elongation at break. The dominance of the carbon nanotube network based on strong interaction forces is also reflected in the wear resistance properties. The wear volume of CNTs-NH_2_/CNTs-COOH/FKM is only 87 mm^3^, which is 29.9% higher compared to the pristine FKM. Additionally, these two strong interfacial interactions resulted in a more robust conductive and thermally conductive network in the FKM matrix, which enhanced the electrical conductivity and thermal conductivity of CNTs-NH_2_/CNTs-COOH/FKM by nine orders of magnitude and 37.0%, respectively. The composite’s thermal conductivity affected by the 10 phr carbon materials is slightly stronger than its own thermal stability, so the initial decomposition temperature of CNTs-NH_2_/CNTs-COOH/FKM slightly decreased by 2 °C compared with the pristine FKM, and the carbon residue rate improved by 293.5%. This effective and mild method provides a reference for the preparation of high-performance fluororubbers and opens up broad application prospects.

## Figures and Tables

**Table 1:** Raw material composition of different FKM nanocomposites.

Sample	FKM	CNTs/FKM	CNTs-COOH /FKM	CNTs-NH_2_/FKM	CNTs-NH_2_/CNTs-COOH/FKM
FKM	100	100	100	100	100
ZnO	3	3	3	3	3
TAIC	3	3	3	3	3
CNTs	-	5	-	-	-
CNTs-COOH	-	-	5	-	5
CNTs-NH_2_	-	-	-	5	5
Trigonox^®^ 101-50D	3	3	3	3	3

## Data Availability

Data available on request from the authors.
